# Retinoic acid receptor-related orphan receptor α reduces lipid droplets by upregulating neutral cholesterol ester hydrolase 1 in macrophages

**DOI:** 10.1186/s12860-020-00276-z

**Published:** 2020-04-22

**Authors:** Hiroshi Matsuoka, Riki Tokunaga, Miyu Katayama, Yuichiro Hosoda, Kaoruko Miya, Kento Sumi, Ami Ohishi, Jun Kamishikiryo, Akiho Shima, Akihiro Michihara

**Affiliations:** 1 Laboratory of Genome Function and Pathophysiology, Faculty of Pharmacy and Pharmaceutical Sciences, Fukuyama University, Fukuyama, Hiroshima, 729-0292 Japan; 2 Laboratory of Biochemistry, Faculty of Pharmacy and Pharmaceutical Sciences, Fukuyama University, Fukuyama, Hiroshima, 729-0292 Japan

**Keywords:** Atherosclerosis, Cholesterol metabolism, Lipid droplets, Macrophages, NCEH1, RORα, Transcriptional regulation

## Abstract

**Background:**

Neutral cholesterol ester hydrolase 1 (NCEH1) catalyzes the hydrolysis of cholesterol ester (CE) in macrophages. Genetic ablation of NCEH1 promotes CE-laden macrophages and the development of atherosclerosis in mice. Dysregulation of NCEH1 levels is involved in the pathogenesis of multiple disorders including metabolic diseases and atherosclerosis; however, relatively little is known regarding the mechanisms regulating NCEH1. Retinoic acid receptor-related orphan receptor α (RORα)-deficient mice exhibit several phenotypes indicative of aberrant lipid metabolism, including dyslipidemia and increased susceptibility to atherosclerosis.

**Results:**

In this study, inhibition of lipid droplet formation by RORα positively regulated NCEH1 expression in macrophages. In mammals, the NCEH1 promoter region was found to harbor putative RORα response elements (ROREs). Electrophoretic mobility shift, chromatin immunoprecipitation, and luciferase reporter assays showed that RORα binds and responds to ROREs in human NCEH1. Moreover, NCEH1 was upregulated through RORα via a phorbol myristate acetate-dependent mechanism during macrophage differentiation from THP1 cells. siRNA-mediated knockdown of RORα significantly downregulated NCEH1 expression and accumulated lipid droplets in human hepatoma cells. In contrast, NCEH1 expression and removal of lipid droplets were induced by RORα agonist treatments and RORα overexpression in macrophages.

**Conclusion:**

These data strongly suggested that NCEH1 is a direct RORα target, defining potential new roles for RORα in the inhibition of lipid droplet formation through NCEH1.

## Background

Neutral cholesterol ester hydrolase 1 (NCEH1), also known as KIAA1363 or arylacetamide deacetylase-like 1, is a key enzyme that suppresses lipid droplet formation by removing cholesterol from macrophage foam cells [1, 2]. In contrast, the ablation of NCEH1 accelerates atherosclerosis by promoting the formation of macrophage foam cells [3, 4]. In addition to cholesterol uptake, the balance of free cholesterol (FC) and cholesterol esters (CEs) is also critical for the regulation of intracellular cholesterol content in macrophage foam cells. After internalization, lipoproteins are localized to late endosomes/lysosomes, where CEs are hydrolyzed into FC by lysosomal acid lipase. To prevent FC release, it is re-esterified on the endoplasmic reticulum by acetyl-CoA acetyltransferase 1 and stored in cytoplasmic lipid droplets. If this pathway is persistently activated, excessive CEs will accumulate in macrophages, thereby resulting in the formation of foam cells. The resulting CEs are hydrolyzed by NCEH1 to release FC for transporter-mediated efflux, which is increasingly recognized as the rate-limiting step in FC outflow [5, 6].

Macrophage-specific overexpression of NCEH1 leads to a significant reduction in atherosclerotic lesions in low-density lipoprotein receptor (LDLR)^−/−^ mice because of enhanced FC efflux and reverse cholesterol transport [3]. NCEH1 enzymatic activity was also shown to be regulated by polyunsaturated fatty acids [7] and paraoxon [8]. NCEH1 expression, which is robust in macrophages and atherosclerotic lesions, has been shown to be regulated by insulin [9] and interleukin (IL)-33 [10]. Moreover, NCEH1 transcripts are downregulated in cortical homogenates from peroxisome proliferator-activated receptor γ coactivator 1-α (PGC-1α)-knockout mice and increased by PGC-1 overexpression [11]. However, the specific mechanisms through which transcriptional regulators modulate NCEH1 expression are still unclear. Notably, two putative response elements for retinoic acid receptor-related orphan receptor α (RORα) are found at − 1451/− 1440 and − 132/− 121 regions upstream of the transcription start site (TSS) in the NCEH1 gene.

The RORα gene encodes a ligand-dependent orphan nuclear receptor that acts as a transcriptional regulator and has been identified as a novel anti-atherosclerosis target gene. RORα regulates target gene expression mainly by binding as monomers to promoter response elements, which typically consist of a consensus AGGTCA half-site preceded by an A/T-rich sequence (ROR response elements [ROREs]) [12]. RORα is constitutively active, meaning that the protein remains in an active conformation in the absence of ligand and that ligand binding can actually suppress receptor activity. Although endogenous ligands of RORα have not yet been fully elucidated, recent evidence suggests that oxygenated sterols might function as high-affinity ligands. Indeed, 7-oxygenated sterols (e.g. 7α-OHC, 7β-OHC, and 7-ketocholesterol), 24-hydroxycholesterol (24-OHC), and 25-hydroxycholesterol (25-OHC) function as inverse agonists for RORα [13, 14]. RORα-deficient mice harboring a natural deletion in the ligand-binding domain exhibit cerebellar ataxia, a phenotype also observed in Staggerer (sg/sg) mutant mice [15], which express mutated RORα and present with vascular dysfunction, dyslipidemia, excessive inflammation, immune abnormalities, and diet-induced atherosclerosis [16–18]. Recent studies have demonstrated decreases in serum and liver triglycerides and total and high-density lipoprotein serum cholesterol in sg/sg mice. These mice also exhibit decreased hepatic expression of sterol regulatory element-binding transcription factor 1 (SREBP-1c) and the reverse cholesterol transporters ABCA1 and ABCG1 [19]. Moreover, RORα positively regulates apolipoprotein A (APOA)-I and APOC-III, suggesting a role in lipid metabolism [20, 21]. The transcriptional activator steroid receptor coactivator-2 (SRC-2) functions as a coactivator with RORα to modulate the expression of the essential gluconeogenesis genes glucose 6-phosphatase (G6Pase) [22] and phosphoenolpyruvate carboxykinase (PEPCK) [23], the rate-limiting enzyme that controls glucose release into the plasma. Moreover, RORα deficiency and treatment with RORα inverse agonists inhibit PEPCK expression and glucose production in mice [24, 25]. Additionally, overexpression of Rev-Erbα, the physiological inhibitor of RORα, suppresses the expression of gluconeogenesis genes in human liver cancer cell lines. Conversely, silencing Rev-Erbα significantly induces the expression of gluconeogenesis-related genes [26–28].

In brain endothelial cells, claudin domain containing 1 (CLDND1), which is involved in tight junction formation, is regulated at the transcriptional level by RORα and at the post-transcriptional level by miR-124 [29, 30]. Moreover, decreased CLDND1 expression in the adult murine cerebellum results in cerebellar hemorrhage [31].

In macrophages, using the CRISPR-Cas9 system, RORα was deleted in human THP1 monocytic cells, and a dramatic increase was observed in the basal expression of a subset of nuclear factor (NF)-κB-regulated anti-inflammatory genes, including tumor necrosis factor, IL-1β, and IL-6, both at the transcriptional and translational levels [32]. RORα is a negative regulator of the inflammatory response, functioning via NF-κB inhibition through IκB activation [33]. Moreover, NF-κB activation requires the removal of IκB from NF-κB through inducible proteolysis, which liberates this transcription factor for migration to the nucleus, where it binds IκB-regulatory elements and induces transcription [34].

As described, RORα is involved in many physiological processes including the regulation of metabolism, development, immunity, and the circadian rhythm. The recent characterization of endogenous ligands for these former orphan nuclear receptors has stimulated the development of synthetic ligands and provided insights into targeting these receptors to treat several diseases including atherosclerosis, diabetes, autoimmunity, and cancer [14, 35, 36]. Nevertheless, the role of RORα in modulating NCEH1 promoter activity is not clear. Accordingly, in this study, the role of RORα in modulating NCEH1 expression was evaluated. The results suggested that the control of NCEH1 expression by synthetic ligands of RORα might facilitate the development of novel anti-arteriosclerosis drugs.

## Results

### Identification of ROREs in the NCEH1 promoter

**Fig. 1:**
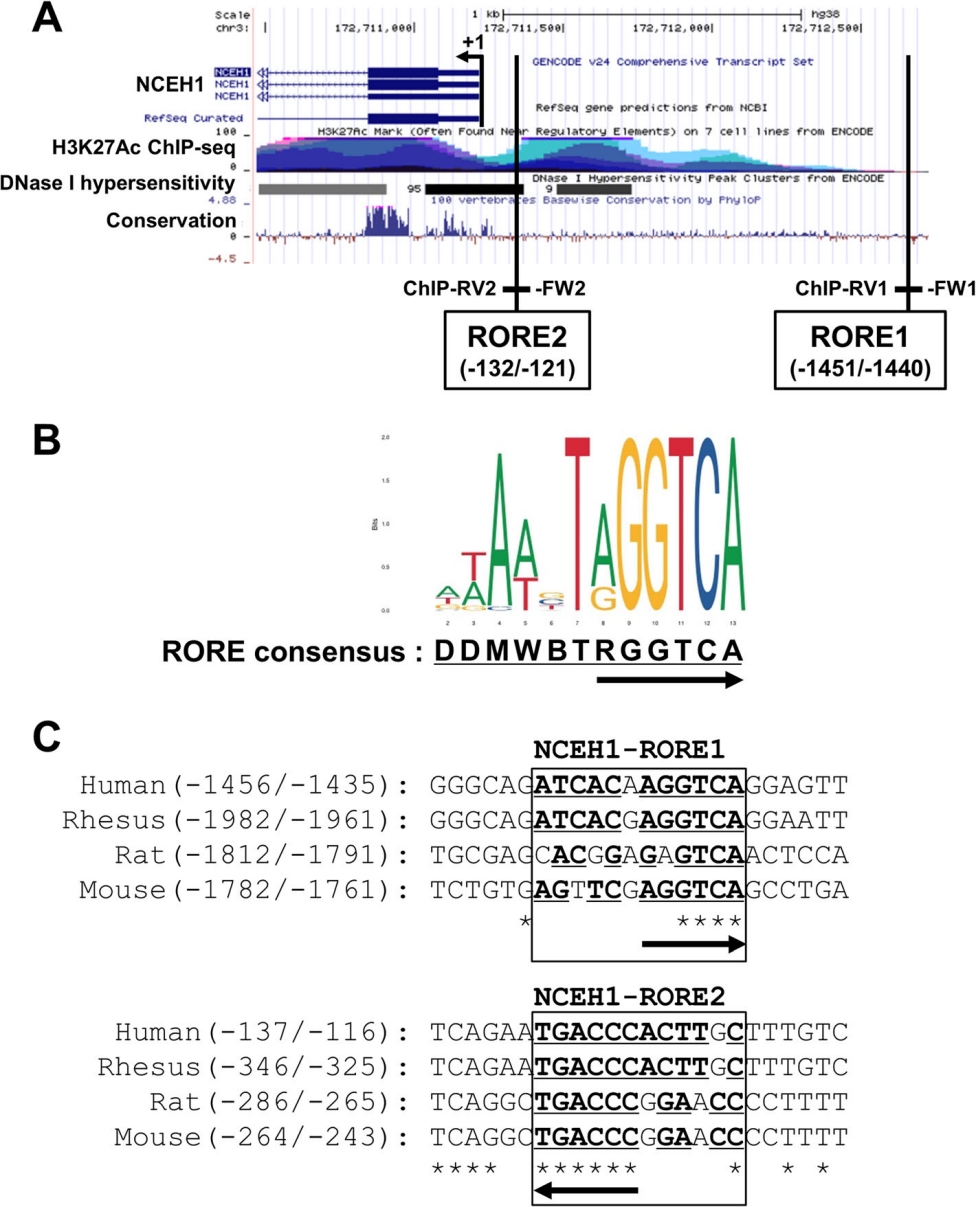
RORα response elements in the NCEH1 promoter region. **a** The UCSC Genome Browser was used to identify distal, conserved, putative RORα response elements (ROREs) in the 5′ region of human NCEH1, which contained the promoter and was located within a DNase I hypersensitivity site. The positions of putative ROREs (RORE1 and RORE2) and transcription start site (TSS, + 1 to position) are indicated by vertical lines. Chromatin immunoprecipitation (ChIP) assays were performed to amplify the ROREs using ChIP-FW1 and ChIP-RV1 or ChIP-FW2 and ChIP-RV2 primer sets. Luciferase reporter constructs of the human NCEH1 promoter region were amplified by PCR using Promoter-FW (− 1689) and Promoter-RV (+ 128) primers. **b** The JASPAR tool was used to identify RORE consensus sequences (i.e. DDMWBTRGGTCA). RORE half-sites were found to be highly conserved regions, as indicated by arrows. **c** Schematic representation of putative ROREs in NCEH1 of various species aligned using ClustalW programs. The human sequence shown includes bases − 1456 to − 1435 as NCEH1-RORE1 and − 137 to − 116 as NCEH1-RORE2 in the putative RORE region, which was tested using electrophoretic mobility shift assays (EMSAs) and reporter experiments. The nucleic acid sequence is shown, with RORE consensus sequences underlined and conserved sequences marked with asterisks in mammals. The direction of the RORE half-site is indicated by arrows

Two putative response elements for RORα were found at − 1451/− 1440 (RORE1) and − 132/− 121 (RORE2) regions upstream of the TSS in NCEH1 (Fig. 1a). The ROREs were found to contain a strongly conserved consensus sequence for RORα-binding sites based on analysis using the JASPER database (http://jaspar.genereg.net/) [37] (Fig. 1b). In particular, the consensus RGGTCA (R: A or G) half-site in NCEH1-ROREs was determined to be highly conserved in various mammals (Fig. 1c).

The ability of RORα to bind its putative response element in the NCEH1 promoter was tested by electrophoretic mobility shift assays (EMSAs) and chromatin immunoprecipitation- polymerase chain reaction (ChIP-PCR). To further identify the RORα-binding site in the NCEH1 gene, H3K27Ac ChIP-sequencing (ChIP-seq) and DNaseI hypersensitivity assays were used as mapping data with the UCSC Genome Browser (http://genome.ucsc.edu/) [38] (Fig. 1a). The location and sequence of this response element are shown in Fig. 1.

**Fig. 2:**
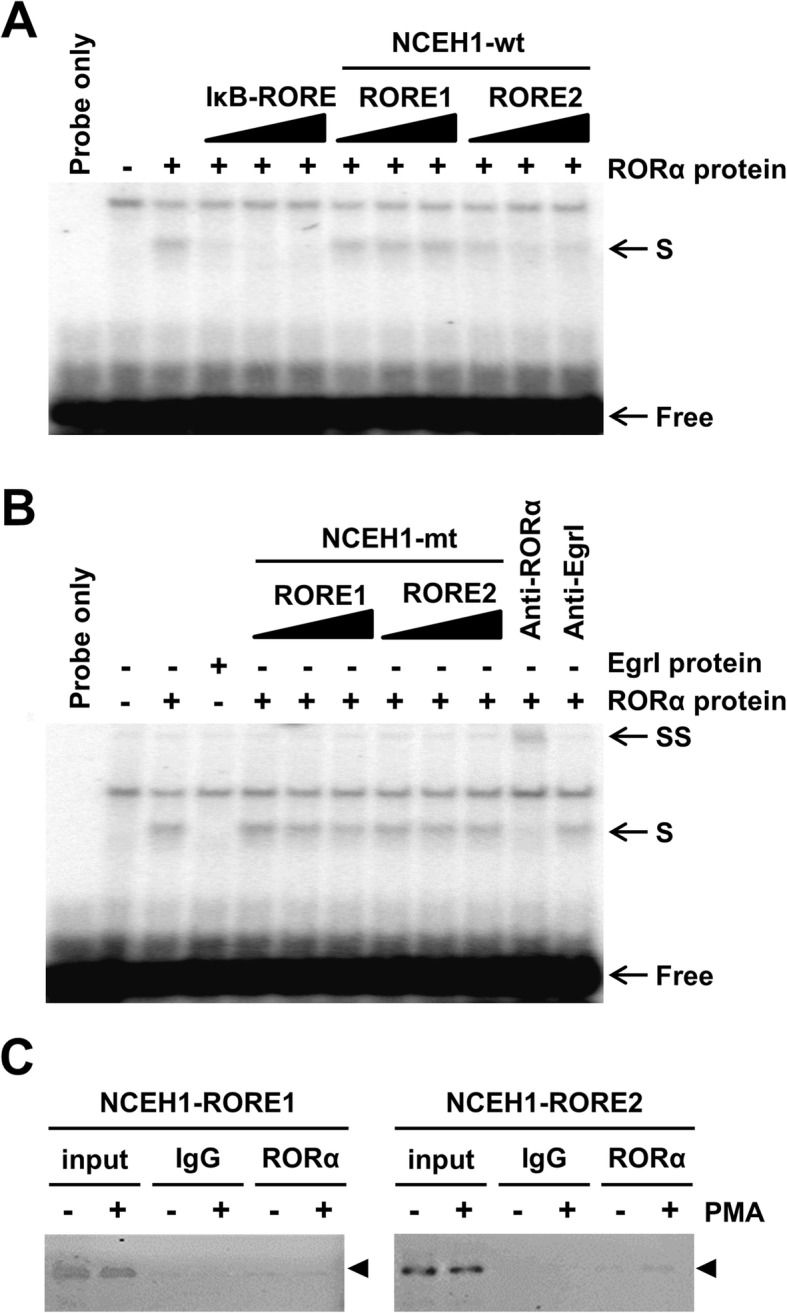
RORα binds a putative response element in NCEH1. **a** Competition of unlabeled duplexes with the labelled IκB RORE probe for binding to in vitro-translated RORα proteins. The IκB probe contained the known RORα response element (RORE) for NF-κB inhibitor α. Reactions containing RORα proteins were carried out in the absence or presence of a 5-, 10-, and 20-fold molar excess of unlabeled duplexes as competitive probes (IκB-RORE, NCEH1-RORE1, and NCEH1-RORE2 [wild-type]). Shifted binding is indicated by S-arrows. The positions of free probes are indicated by free arrows. **b** Electrophoretic mobility shift assays (EMSAs) were used to test the ability of unlabeled mutated probes (5-, 10-, and 20-fold molar excess) to relieve the inhibition of binding of RORα to the putative response element (S-arrows). Anti-RORα antibodies were pre-incubated with RORα protein before adding the labeled probe for the formation of the super-shift band (SS-arrows). Negative control experiments were performed using EgrI protein and antibodies. Lane 1: labelled probe only; lane 2: reaction containing crude products from in vitro translation; lane 3: probe incubated with crude product obtained from in vitro translation in the absence of the RORα expression vector. **c** Chromatin immunoprecipitation (ChIP) assays were performed using chromatin isolated from human monocytes and differentiated macrophages treated with 100 nM phorbol 12-myristate 13-acetate (PMA) for 24 h. Crosslinked cell lysates were immunoprecipitated with rabbit IgG (IgG) or polyclonal anti-RORα-specific antibodies (RORα). DNA precipitates were isolated and then subjected to PCR using primer pairs covering either RORE1 or RORE2 of the NCEH1 promoter region. Control PCR was performed with non-immunoprecipitated genomic DNA (input)

In addition to conventional EMSA, a 20-bp fragment spanning positions − 132 to − 121 and − 1451 to − 1440 of the NCEH1 promoter was generated as a cold probe and competitor for hot probes, end-labeled similar to IκB, which contains a known RORE, and incubated with RORα obtained by in vitro translation (Fig. 2a). RORα-depended sequence-specific mobility shifts were inhibited by the addition of excess unlabeled probe such as IκB and wild-type NCEH1-RORE (RORE1 as a weak binding site and RORE2 as a strong binding site), but not by mutant NCEH1-RORE. Additionally, anti-RORα antibodies suppershifted as bound to the formation of the DNA–protein complex, suggesting that RORα was present in the DNA–protein complex. In contrast, addition of anti-early growth response protein I (EgrI) antibodies as a negative control resulted in not bound to the DNA–protein complex, so RORα-depended binding manner (Fig. 2b).

To further validate the transactivation ability of RORα at the NCEH1 promoter, ChIP assays were performed. The chromatin fragments of the NCEH1 promoter region from − 1508 to − 1257 and from − 260 to − 14, containing the RORE1 and RORE2 sites, were immunoprecipitated by anti-RORα antibodies (Fig. 2c). As a control, the addition of IgG alone did not result in immunoprecipitation of the chromatin fragment of the NCEH1 promoter (Fig. 2c). Additionally, quantitative PCR with ChIP samples revealed an increase of 1.2-fold in relative binding intensity to RORE1 in differentiated cells treated with phorbol 12-myristate 13-acetate (PMA) and an increase of 7.7-fold in binding intensity to RORE2 as compared to that in macrophages (data not shown).

### Characterization of ROREs in the NCEH1 promoter

**Fig. 3:**
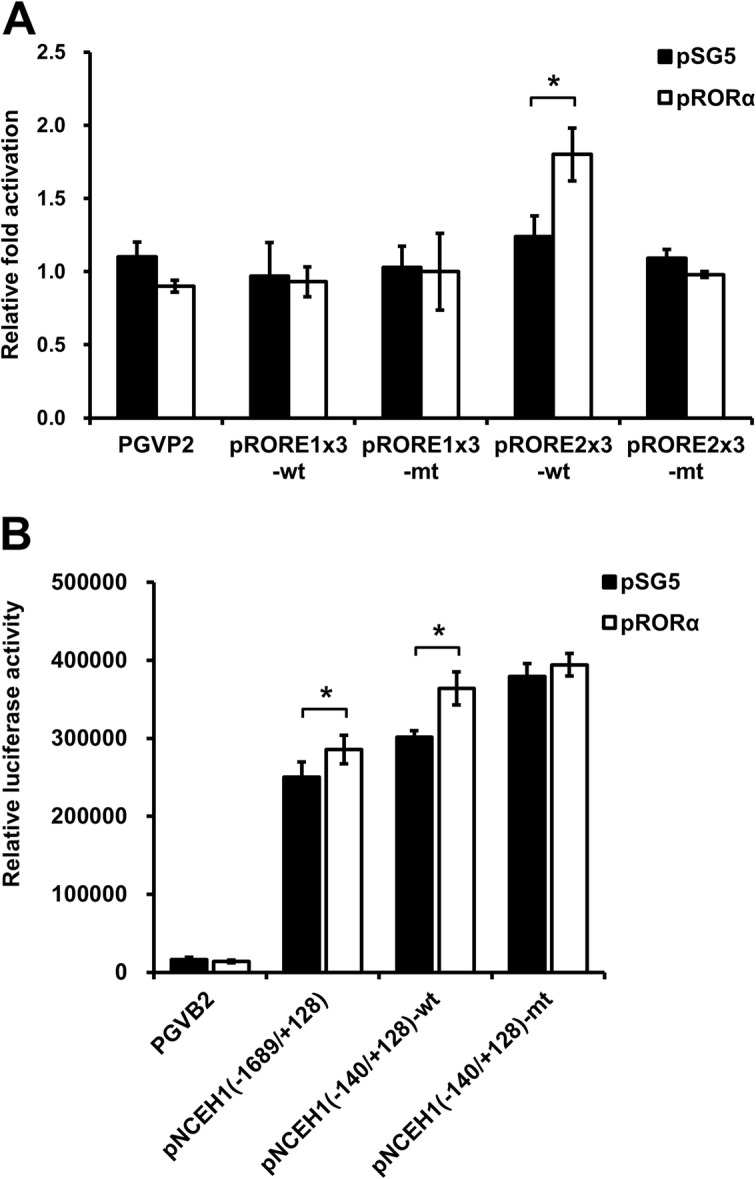
RORα response elements (ROREs) of the NCEH1 promoter are directly activated by RORα. **a** HEK293 cells were transiently transfected with the luciferase reporter plasmid in the presence of pSV-βgal. As a luciferase reporter plasmid, wild-type (wt) or mutant (mt) ROREs with three direct repeats, as found in the NCEH1 promoter region, were used. At 36 h after transfection, HEK293 cells were co-transfected without (pSG5) or with RORα expression vector (pRORα). Luciferase activity in the cell lysates was determined and expressed as fold-change in RORα activation based on luciferase activity. Data are means ± standard errors (*n* = 4). *, *p* < 0.05 versus PGVP2 control vector. **b** HEK293 cells were transfected without (pSG5) or with RORα expression vector (pRORα) plus luciferase-driven wild-type [pNCEH1(− 1689/+ 128) or pNCEH1(− 140/+ 128)-wt] or mutant [pNCEH1(− 140/+ 128)-mt] NCEH1 promoters. Mutations in the promoter included the mutation of RORE2. Data are fold-changes in transactivation relative to basal activity and are reported as means ± standard errors (*n* = 4). *, *p* < 0.05

In HEK293 cells, luciferase reporter assays were utilized to assess the activity of the putative RORE1 and RORE2 in the NCEH1 promoter. We used the UCSC Genome Browser to identify distal, conserved, putative ROREs in the 5′ region of the NCEH1 gene, which contained a promoter located within a DNase I hypersensitivity site. ChIP-seq assays indicated that acetylation of lysine 27 in histone H3 might be involved in enhancing transcription (Fig. 1a and b). Luciferase expression after co-transfection of the RORα expression plasmid with luciferase response plasmids containing three copies of the ROREs (pRORE1x3-wt, pRORE1x3-mt, pRORE2x3-wt, and pRORE2x3-mt) resulted in 0.93-, 1.00-, 1.80-, and 0.98-fold activation, respectively, compared to that with the empty control vector (PGVP2) containing the SV40 promoter (Fig. 3a). Moreover, compared to the response of NCEH1-RORE, IκB-RORE, a known target gene of RORα, was found to increase 2.2-fold with two direct RORE repeats and to increase by 1.3-fold with one RORE observed (data not shown). Furthermore, the region from − 1689 to + 128 of the NCEH1 gene was cloned into the luciferase reporter vector PGVB2 and transiently transfected into the human embryonic kidney cell line HEK293. Luciferase expression from pNCEH1(− 1689/+ 128), which contained two ROREs (RORE1 and RORE2), and pNCEH1 (− 140/+ 128)-wt was increased along with the transient overexpression of RORα (Fig. 3b). In contrast, pRORE1x3-mt, pRORE2x3-mt, and pNCEH1(− 140/+ 128)-mt were not activated. These results suggested that RORα activates NCEH1 expression at the ROREs because a binding site was required in the NCEH1 promoter region.

### RORα overexpression induces NCEH1 expression in HEK293 cells

**Fig. 4:**
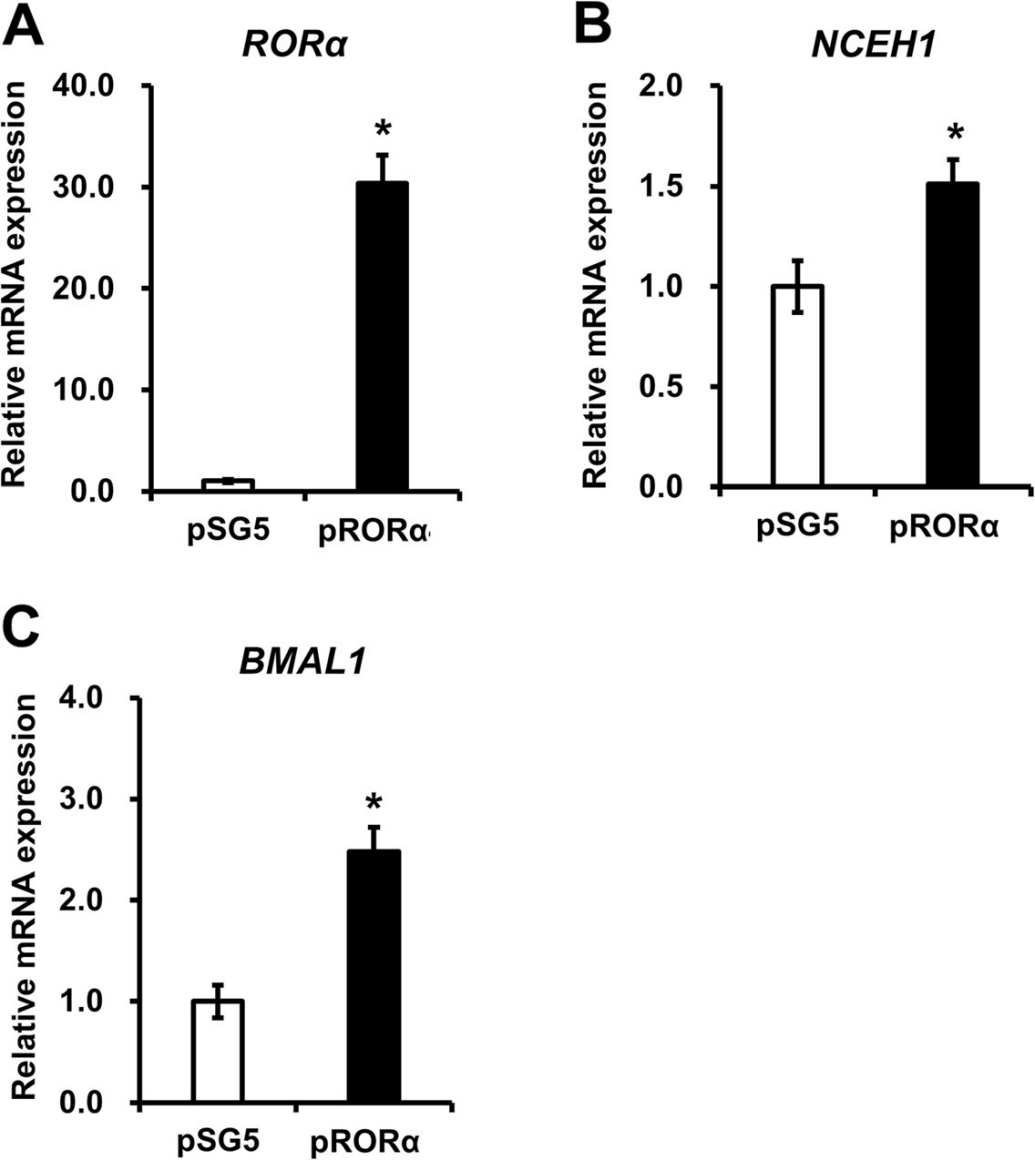
Effects of RORα overexpression on NCEH1 expression. Transient overexpression of RORα was performed in HepG2 cells by transfecting cells with empty vector (pSG5) or with RORα expression vector (pRORα) for 48 h. RORα (**a**), NCEH1 (**b**), and BMAL1 (**c**) gene expression levels were evaluated. The expression levels of RORα-target genes induced by pRORα are presented as the fold-change relative to those with pSG5. Data are means ± standard errors (*n* = 4). *, *p* < 0.05

To examine whether NCEH1 expression increases with RORα expression, a transient RORα overexpression was carried out. Analyses were performed in HEK293 cells treated for 48 h with the RORα expression vector (pRORα) or the vehicle vector (pSG5). The mRNA levels of RORα were increased in HEK293 cells transfected with pRORα (Fig. 4a). Moreover, NCEH1 expression levels were increased following pRORα transfection (Fig. 4b). Additionally, the expression levels of BMAL1, as a positive control RORα-target gene, were increased (Fig. 4c). RORα was found to positively regulate the expression of NCEH1 in HEK293 cells.

### RORα and NCEH1 expression increase in macrophages at both the mRNA and protein levels

**Fig. 5:**
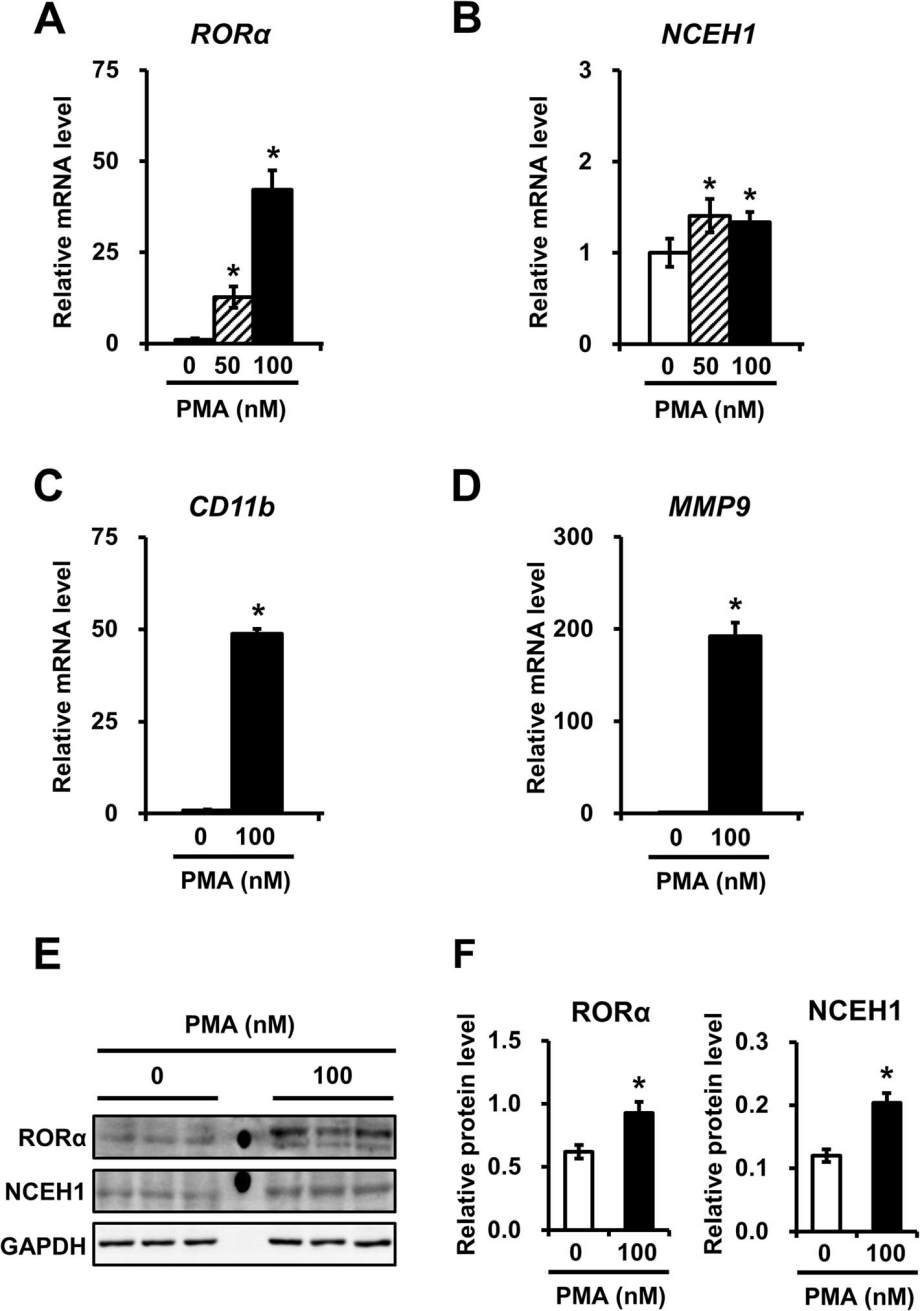
NCEH1 expression is altered in a phorbol 12-myristate 13-acetate (PMA)-dependent manner during macrophage differentiation in THP1 cells. **a**–**d** THP1 cells were treated with 50 nM (shaded bars), 100 nM (closed bars), or no PMA (open bars) for 12 h. mRNA expression of RORα (**a**), NCEH1 (**b**), CD11b (**c**), and MMP9 (**d**) was analyzed by qRT-PCR, and data were normalized to 18S rRNA levels. Data are means ± standard errors (*n* = 4). *, *p* < 0.05. **e** THP1 cells were treated with or without 100 nM PMA for 24 h. Protein expression of RORα, NCEH1, and GAPDH was analyzed by immunoblot analysis. **f** Summary of RORα and NCEH1 protein levels for each treatment. Data were normalized to GAPDH protein expression. Data are means ± standard errors (*n* = 4). *, *p* < 0.05

In macrophages, RORα increased the mRNA and the protein expression of NCEH1. To confirm this finding under our experimental conditions, THP1 cells were treated with 50 or 100 nM PMA to induce the differentiation of macrophages. Real-time PCR analysis showed that the expression of RORα and NCEH1 were increased together with PMA induction in THP1 cells at 12 h (Fig. 5a and b). Similarly, western blot analysis showed that RORα expression and NCEH1 protein expression were increased in THP1 cells at 24 h (Fig. 5e and f). Moreover, macrophage differentiation was monitored by measuring cluster of differentiation (CD) 11b and matrix metalloproteinase (MMP) 9 expression, as markers of macrophages (Fig. 5c and d). The results demonstrated that RORα expression increased along with endogenous NCEH1 expression at both the mRNA and protein levels in THP1 macrophages.

### siRNA targeting of RORα reduces NCEH1 expression and lipid droplet formation

**Fig. 6:**
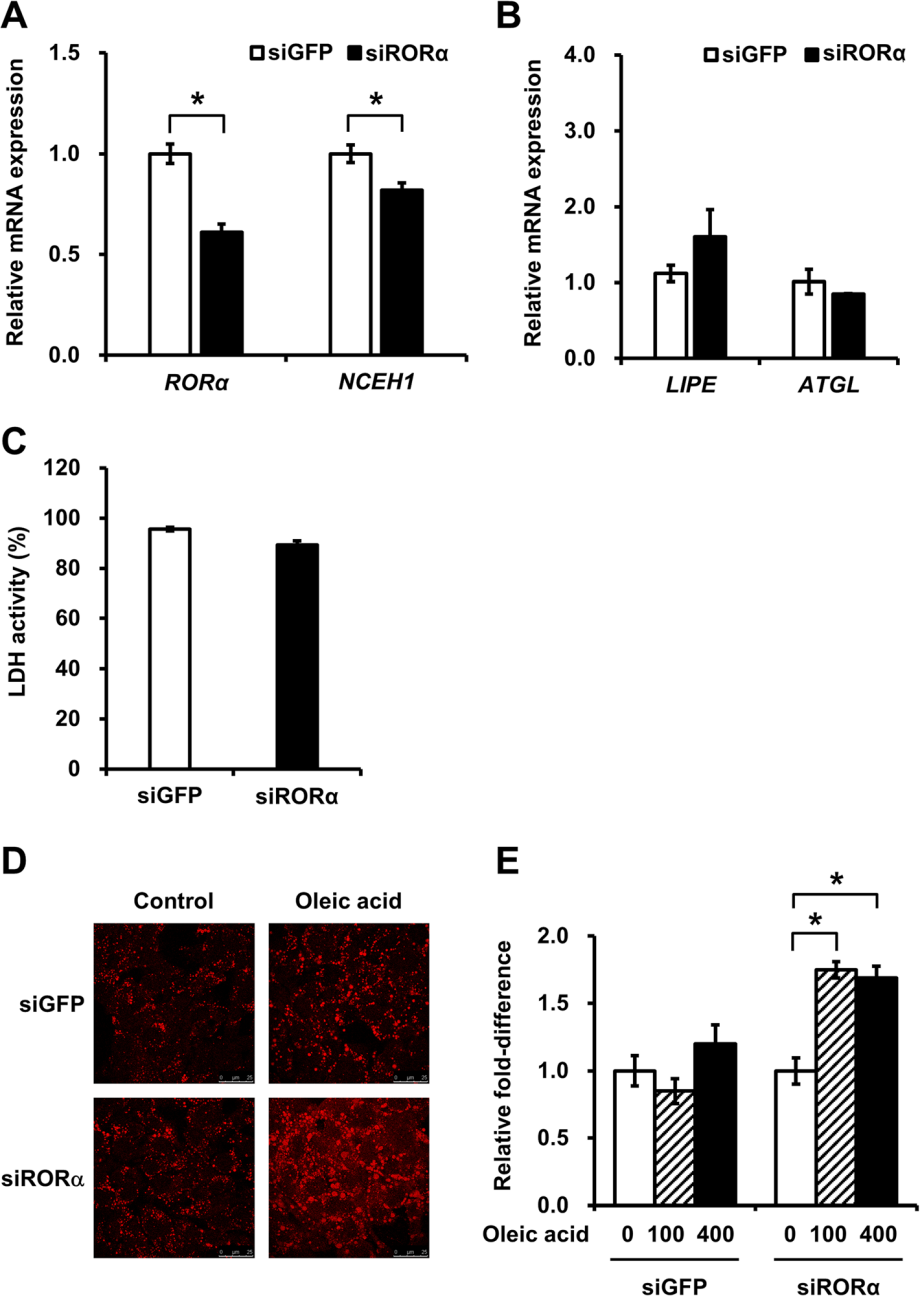
Effects of RORα deficiency on NCEH1 and lipase expression and lipid droplet accumulation. **a** HepG2 cells were transfected with 50 nM siRNA and analyzed by qRT-PCR to measure the expression of RORα and NCEH1. siRNAs targeting sequences in the RORα coding region (siRORα) were used and the resulting RORα and NCEH1 mRNA levels were measured. siRNA targeting green fluorescent protein was used as a negative control (siGFP). Data are means ± standard errors (*n* = 3) and were normalized to 18S rRNA levels. *, *p* < 0.05. **b** Effects of siRNA transfection on the expression of adipose triglyceride lipase (ATGL) and Lipase E (LIPE). **c** Effects of siRNA transfection on cell viability were estimated by measuring lactate dehydrogenase (LDH) activity in the culture medium of siRNA-transfected cells. Data are means ± standard errors (*n* = 4). *, *p* < 0.05. **d** Lipid droplet accumulation was demonstrated by Oil Red O staining. At 48 h after transfection with siRNA targeting RORα or GFP, HepG2 cells were treated with 400 μM oleic acid or DMSO (as a control) for 24 h. **e** HepG2 cells were transfected with siRNA and treated with 0, 100, or 400 μM oleic acid for 24 h. Subsequently, the quantification of absorbance was performed to determine Oil Red O staining levels in the cells. Data are means ± standard errors (*n* = 3). *, *p* < 0.05

To further investigate the effect of RORα knockdown, we determined whether this could block the accumulation of lipid droplets through the downregulation of NCEH1 expression in HepG2 cells. Indeed, siRNA targeting the sequences around 258 and 1388 bp downstream of the RORα start codon suppressed NCEH1 transcription to 82%, compared to 100% transcription in siGFP-transfected cells (Fig. 6a). Negative control siRNA (siGFP, targeting green fluorescent protein) did not affect NCEH1 transcription. No changes were observed in the expression of adipose triglyceride lipase (ATGL) and Lipase E (LIPE, Fig. 6b). The effects of siRNA transfection on cell viability were estimated by measuring lactate dehydrogenase (LDH) activity at 48 h after transfection with siRORα. The results showed that LDH activities in siGFP- and siRORα-transfected cells (95.6 and 89.3%, respectively; Fig. 6c) were similar to those in untreated cells. Subsequently, cells were treated with oleic acid for 24 h, and Oil Red O staining was performed. Notably, lipid droplet accumulation in cells transfected with siRORα was markedly increased compared with that in negative controls (fold-changes in cells transfected with siGFP at 100 and 400 nM: 1.75 and 1.69, respectively; Fig. 6d and e).

### Effects of agonist-induced RORα activation on NCEH1 expression

**Fig. 7:**
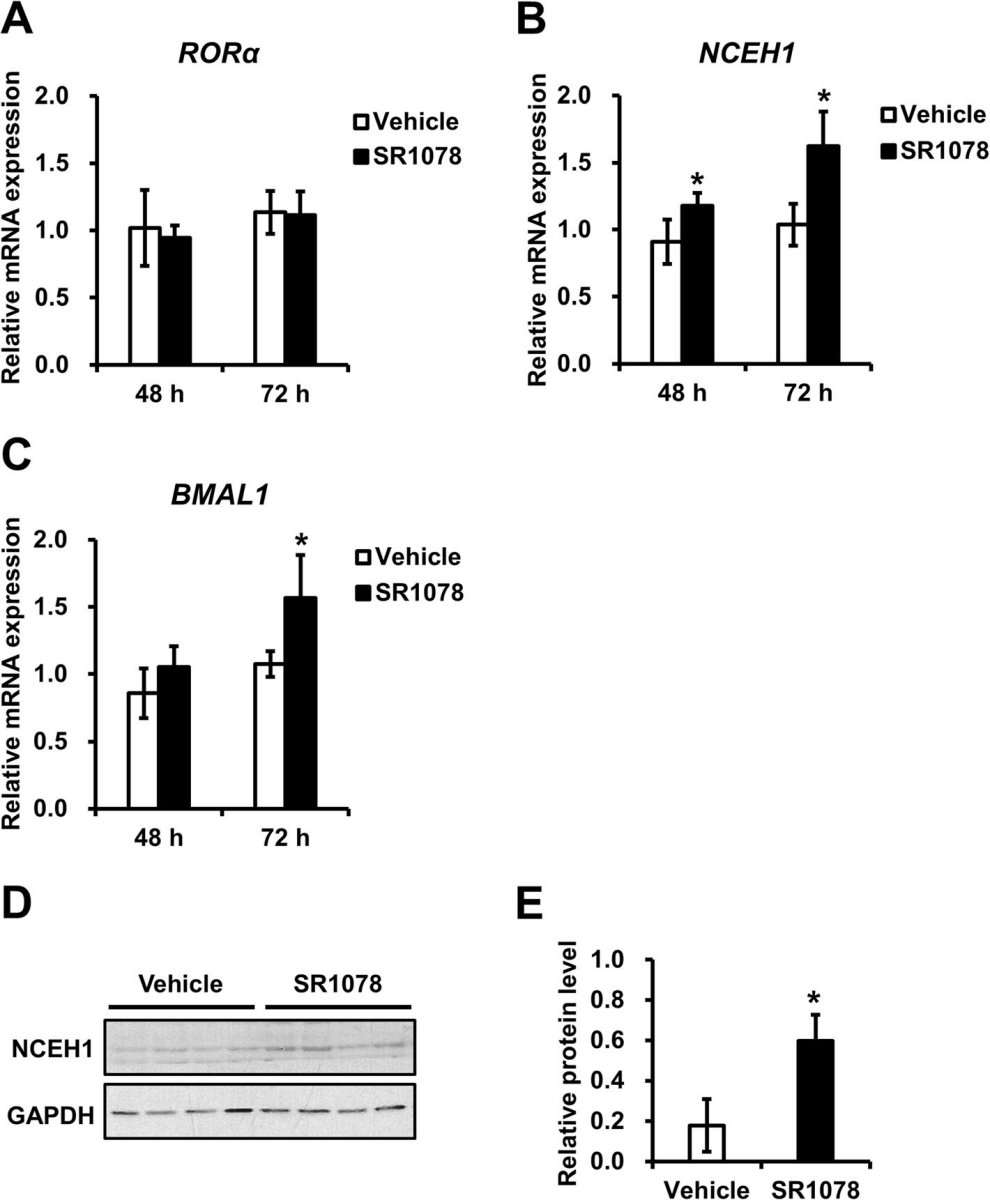
Effects of agonist-induced RORα activation on NCEH1 expression. HepG2 cells expressing endogenous RORα were treated without (Vehicle, open bars) or with 5 μM SR1078 (closed bars) for 48 or 72 h, and RORα (**a**), NCEH1 (**b**), and BMAL1 (**c**) gene expression levels were evaluated. The expression levels of RORα-target genes stimulated by the RORα agonist are presented as the fold-change relative to those with the vehicle. Data are means ± standard errors (*n* = 3). *, *p* < 0.05. **d** THP1 cells were treated with 100 nM phorbol 12-myristate 13-acetate (PMA) for 72 h and then treated without or with 5 μM SR1078 for 24 h. Protein expression of NCEH1 and GAPDH was analyzed by immunoblotting. **e** Summary of NCEH1 protein levels with each treatment. Data were normalized to GAPDH protein expression. Data are means ± standard errors (*n* = 4). *, *p* < 0.05

The effect of inducing NCEH1 expression by an RORα agonist was examined. Analyses were performed in HepG2 cells treated for 48 or 72 h with 5 μM SR1078 (RORα agonist) or vehicle (DMSO). The mRNA levels of RORα were not altered in HepG2 cells treated with 5 μM SR1078 (Fig. 7a). In contrast, NCEH1 expression levels were increased following treatment with an RORα agonist (Fig. 7b). Additionally, the expression levels of BMAL1, as a positive control RORα-target gene, were increased (Fig. 7c). In PMA-differentiated THP1 macrophages treated for 24 h with 5 μM SR1078 (RORα agonist) or vehicle (DMSO), the protein levels of NCEH1 were increased following treatment with an RORα agonist (Fig. 7d and e).

### RORα activation inhibited lipid accumulation in macrophages induced by oxidized-LDL

**Fig. 8:**
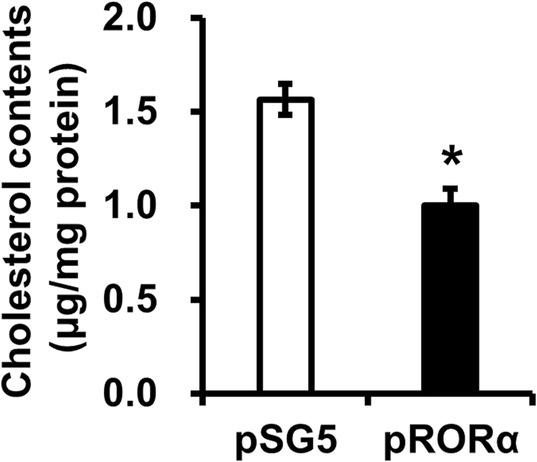
Effects of RORα overexpression on intracellular cholesterol contents in macrophages. RAW264.7 cells were transfected with empty vector (pSG5) or RORα expression vector (pRORα), and cultured with 10 μg/mL of ox-LDL for the formation of macrophage foam cells. Cells were extracted and measured intracellular cholesterol content. Data are represented as means ± standard errors (*n* = 3). *, *p* < 0.05. Ox-LDL, oxidized low density lipoprotein

In order to investigate the effect of RORα activation on lipid accumulation, the intracellular cholesterol levels after RORα overexpression, were measured in foam cells treated with oxidized low density lipoprotein (ox-LDL) in RAW264.2 macrophages. RAW264.7 cells transfected with the RORα expression vector for 48 h, were analyzed and the results were compared to that of the empty vector. RORα overexpression resulted in a decrease in the total cholesterol levels (Fig. 8).

## Discussion

NCEH1 is a key enzyme that suppresses lipid droplet formation by removing cholesterol in macrophage foam cells [1, 2]. Downregulation of NCEH1 promotes atherosclerosis by increasing the generation of macrophage foam cells [3, 4]. Macrophage-specific overexpression of NCEH1 leads to a significant reduction in the atherosclerotic lesion area in LDLR^−/−^ mice because of enhanced FC efflux and reverse cholesterol transport [3]. RORα-deficient mice harboring a natural deletion in the ligand-binding domain exhibit cerebellar ataxia, a phenotype also observed in sg/sg mice [15], which express mutated RORα and present with dyslipidemia, excessive inflammation, immune abnormalities, vascular dysfunction, and diet-induced atherosclerosis [16–18]. In humans, RORα is highly expressed in normal arterial cells. However, RORα expression is significantly reduced in atherosclerotic plaques [39]. The RORα expression level and the malignancy of arteriosclerosis may be closely related.

In this study, the results showed that the NCEH1 gene is a direct target of RORα in macrophages and is related to lipid droplet regulation. First, a global search for ROREs as RORα-binding sites involved in transcriptional regulation within − 1500 to + 500 from the TSS of human genes in the DBTSS database [40] and by TFBIND [41] analysis as software for searching transcription factor binding sites on DNA sequences identified the promoter region of NCEH1 as a putative target of RORα. Therefore, in this study, the promoter region of NCEH1 was isolated and characterized. Using ENCODE data, two distal, conserved, putative ROREs were identified in the 5′ region of the NCEH1 gene containing the promoter and a highly conserved RGGTCA (R: A or G) half-site motif. One of the ROREs, RORE2, was located within a DNase I hypersensitivity site, and H3K27Ac ChIP-seq indicated transcriptional enhancement at RORE2, but not at RORE1. A comparison of the human RORE with those of rhesus monkeys, rats, and mice revealed that this element is highly conserved between humans and rhesus monkeys. However, low sequence conservation was observed with rats and mice, suggesting that this element might not have a major role in the transcriptional regulation of RORα in these animals. The results also showed that RORα binds this response element and that the receptor modestly stimulates expression. In addition, PMA-induced stimulation of NCEH1 expression was dependent on RORα induction in macrophages, and the transient overexpression of RORα induced NCEH1 transcription. Moreover, the suppression of RORα expression by siRNA significantly decreased NCEH1 transcription and accumulated lipid droplet. Additionally, RORα overexpression and RORα agonist-treated cells showed an increase in the NCEH1 expression and a decrease in the intracellular cholesterol content in macrophages. Taken together, these data strongly indicated that NCEH1 is a direct target of RORα, defining potential new roles for RORα in the inhibition of lipid droplet formation through NCEH1.

Macrophages from RORα-deficient sg/sg mice show the ability to accumulate lipids and therefore harbor large lipid droplets. Bone marrow-derived macrophages from sg/sg mice exhibit significantly reduced mRNA and protein levels of cholesterol 25-hydroxylase (Ch25h) and deficiencies in phagocytosis. Ch25h produces 25-OHC from cholesterol; 25-OHC functions as an agonist for liver X receptor α (LXRα) and is an inverse agonist for RORα, functioning through the transcriptional regulation of target genes [42, 43]. Recent work has also defined a role for LXRs in the regulation of gene expression in response to cellular lipid loading. ABCA1 and ABCG1, two members of the ATP-binding cassette (ABC) family of transporter proteins, are highly induced in lipid-loaded macrophages [44, 45]. LXRs activate target genes by binding DNA sequences associated with target genes. LXRs then bind consensus elements (LXREs) as heterodimers with isoforms of retinoid X receptors. LXRE consists of two direct repeats of the consensus sequence AGGTCA separated by four nucleotides [46, 47]. In contrast, RORα regulates target gene expression mainly by binding as monomers to promoter response elements, which typically consist of a consensus AGGTCA half-site preceded by an A/T-rich sequence [12]. The response elements on NCEH1 are not two direct repeats but are instead consensus ROREs involved in direct transcriptional regulation by RORα. RORα has been shown to directly regulate hepatic expression of SREBP-1c and the reverse cholesterol transporters ABCA1 and ABCG1 [19]. Moreover, RORα positively regulates APOA-I and APOC-III, suggesting a role for the receptor in lipid metabolism [20, 21] and lipid homeostasis [48, 49]. Additionally, the transcriptional activator SRC-2 functions as a coactivator with RORα to modulate G6Pase [22] and PEPCK expression [23]. Thus, RORα and LXR share many common target genes. In macrophages, RORα and 25-OHC crosstalk regulates lipid droplet homeostasis, whereas the absence of NCEH1 augments 25-OHC-induced endoplasmic reticulum stress and apoptosis [50, 51]. 25-OHC functions as an oxysterol to regulate RORα activation and NCEH1 expression. Moreover, the treatment of liver macrophages with synthetic RORα ligands was found to modulate nonalcoholic steatohepatitis (NASH); activation of RORα by SR1078 [52] as an RORα agonist results in protection against NASH and loss of RORα function, whereas inhibition of RORα function by SR3335 [25], an RORα-selective inverse agonist, results in the progression of NASH [53]. Further pharmacological and pathophysiological studies are currently investigating the development of candidate RORα ligands for various applications [54, 55].

In pharmacological therapies involving nuclear receptor ligands, increased activity of lipoprotein lipase (LPL) in response to various peroxisome proliferator-activated receptors (PPARs) might explain the hypotriglyceridemic effects of fibrates, thiazolindinediones, and fatty acids, which are known activators (and/or ligands) of the various PPARs. Treatment with compounds that preferentially activate PPARα, such as fenofibrate, induces LPL expression exclusively in the livers of rats. In addition, the antidiabetic thiazolidinedione, a high-affinity ligand for PPARγ, has no effect on the liver but induces LPL expression in rat adipose tissues [56]. Moreover, endocrine therapy targeting the estrogen receptor (ER) is a standard of care for the treatment of postmenopausal women with ER-positive breast cancer. Given the dependence of these tumors on active ER signaling, the predominant treatment strategy has been to inhibit various aspects of this pathway, including directly antagonizing the ER with the use of selective ER modulators. Selective ER modulators have been used for the treatment of advanced breast cancer and are currently being evaluated for all stages of ER-positive disease [57]. Selective receptor modulators (SRMs) are receptor ligands that exhibit agonistic or antagonistic biocharacteristics in a cell- and tissue context-dependent manner. SRM-induced alterations in the conformation of the ligand-binding domains of nuclear receptors influences their abilities to interact with other proteins such as coactivators and corepressors [58]. In contrast, RORα ligands might be able to control some RORα-target genes, although it is currently not possible to achieve pathological tissue-specific selectivity.

## Conclusions

In summary, the data from this study suggested that human NCEH1 is a direct target of RORα, containing two functional response elements in the NCEH1 promoter region. Our results defined potential new roles for RORα in the cholesterol metabolism of macrophages, possibly by regulating NCEH1 expression. NCEH1 activation, which leads to the removal of cholesterol esters in lipid droplets in macrophages, may be important for the suppression of arteriosclerosis. Additionally, preventing the accumulation of lipid droplets using agonists of RORα is a possible new therapeutic approach. Improving our understanding of the interactions between RORα and its ligands may facilitate the development of specific drugs for the treatment of inflammation, metabolic diseases, obesity, and atherosclerosis.

## Methods

### Cell culture

Human THP1 monocytic cells were used as a model for monocytes and were maintained in RPMI-1640 supplemented with 10% fetal bovine serum (FBS), 100 U/mL penicillin, and 100 μg/mL streptomycin. THP1 cells were differentiated into macrophages following treatment with 100 nM PMA for 12 or 24 h. RAW264.7 macrophages, HEK293 embryonic kidney cells and HepG2 hepatoma cells were maintained in Dulbecco’s modified Eagle’s medium (D-MEM) supplemented with 10% FBS, 100 U/mL penicillin, and 100 μg/mL streptomycin. All cell lines were obtained from the American Type Culture Collection (Manassas, VA, USA).

### EMSA

EMSAs were performed as described previously [23]. The IκB probe was prepared by radiolabeling synthetic double-stranded DNA using [*γ*-^32^P] ATP (Perkin-Elmer, Waltham, MA, USA). Unlabeled probe was used as a competitive inhibitor, and IκB, which contained a known RORE [33], was used as a positive competitive control. In this assay, 0.02 pmol ^32^P-radiolabeled probe was incubated with RORα crude product from in vitro translation, along with 0.1 and 0.4 pmol unlabeled DNA as wild-type and mutant NCEH1-RORE1 and -RORE2. Probe sequences are listed in Supplementary Table S1. For supershift assays, anti-RORα antibodies (cat. no. sc-28,612; Santa Cruz Biotechnology, Santa Cruz, CA, USA) or anti-EgrI antibodies (cat. no. sc-110; Santa Cruz Biotechnology) as a negative control were pre-incubated with the RORα crude product prior to the addition of the oligonucleotide probe. Gel electrophoresis was conducted at 120 V using 4% native polyacrylamide gels and 0.5× TBE buffer. The autoradiograms were obtained and quantified using a Typhoon 9400 variable mode imager (GE Healthcare, Little Chalfont, UK).

### ChIP assays

ChIP assays were performed using a OneDay ChIP Kit (Diagenode, Liege, Belgium). THP1 monocytes and differentiated macrophages were used as samples. Samples containing protein/chromatin complexes were incubated with antibodies specific for RORα (cat. no. sc-28,612; Santa Cruz Biotechnology) or nonimmunized IgG as a negative control overnight at 4 °C. Immunoprecipitated complexes were eluted with elution buffer (1% sodium dodecyl sulfate [SDS], 50 mM tris-HCl [pH 7.5], and 10 mM ethylenediaminetetraacetic acid). Sample DNA was purified and amplified by PCR using designed primers specific to the NCEH1 promoter region. Primer sequences are listed in Supplementary Table S1. The band intensities of PCR products were analyzed with a CS Analyzer (Atto, Tokyo, Japan). The relative binding intensity to ROREs was calculated by fixed quantitative PCR using a Light Cycler 480 SYBR Green I Master kit (Roche Diagnostics, Mannheim, Germany) in a Light Cycler 480 instrument (Roche).

### Luciferase reporters

The luciferase reporter plasmids pRORE1x3-wt, pRORE2x3-wt, pRORE1x3-mt, and pRORE2x3-mt containing triplet repeats of NCEH1-ROREs were constructed. Synthetic oligonucleotides corresponding to sense and antisense ROREs, as used for EMSA (Supplementary Table S1), were phosphorylated with T4 DNA polynucleotide kinase (Takara Bio, Shiga, Japan), mixed, and annealed. Each resulting double-stranded oligonucleotide was cloned into the SmaI site of the reporter vector PGVP2 (Nippon Gene, Tokyo, Japan) containing the SV40 promoter. Moreover, the human NCEH1 promoter [pNCEH1(− 1689/+ 128)], from − 1689 to + 128 relative to the TSS, was amplified by PCR and inserted as a KpnI/MluI-fragment into the promoterless luciferase expression vector PGVB2 (Nippon Gene). The wild-type and mutant promoter sequences for RORE2 [pNCEH1(− 140/+ 128)-wt and -mt] were generated by PCR using the pNCEH1(− 1689/+ 128) plasmid as a template. Briefly, the primer sets Promoter-RORE2-FW-KpnI, Promoter-RORE2mt-FW-KpnI, and Promoter-RV-MluI were synthesized to incorporate the desired mutation (Supplementary Table S1), and a continuous fragment was produced. This fragment was then cloned as a KpnI/MluI-fragment into PGVB2. All cloned plasmids were purified using a Qiagen Plasmid Mini Kit (Qiagen, Valencia, CA, USA). Inserts were confirmed by sequencing using PGVB2-FW and PGVB2-RV primers.

### Transfection and luciferase activity assay

HEK293 cells were transfected using Lipofectamine 2000 (Life Technologies, Gaithersburg, MD, USA). Briefly, cells were seeded at 1 × 10^5^ cells/well in D-MEM containing 10% fetal calf serum. After 1 day at 37 °C and 5% CO_2_, cells in each well were transfected for 16 h with a mixture of 100 ng various luciferase reporter plasmid, 100 ng RORα expression plasmid (pRORα) or empty plasmid (pSG5), plus 100 ng β-galactosidase reporter plasmid (pSV-βgal), which was used to normalize luciferase activity. Cells were grown for an additional 24–32 h in fresh medium and finally lysed. The lysate was assayed for luciferase activities using a PicaGene Luminescence Kit (Toyo Inc., Tokyo, Japan) with a Luminescencer-PSN AB-2200 (Atto). Data were collected from at least four independent experiments.

### Quantitative reverse transcription-PCR (qRT-PCR)

THP1 cells were seeded at 1 × 10^5^ cells/well in RPMI-1640 supplemented with 10% FBS, 100 U/mL penicillin, and 100 μg/mL streptomycin. After 24 h at 37 °C and 5% CO_2_, THP1 cells were differentiated into macrophages via treatment with 100 nM PMA for 12 h. In RORα agonist-treated experiments, THP1 cells were differentiated into macrophages by treating them with 100 nM PMA for 72 h and were then treated with 5 μM SR1078, an RORα agonist, for 24 h. HepG2 cells were seeded at 1 × 10^5^ cells/well in D-MEM supplemented with 10% FBS, 100 U/mL penicillin, and 100 μg/mL streptomycin. After 24 h at 37 °C and 5% CO_2_, HepG2 cells were induced by treatment with 5 μM SR1078 as an RORα agonist for 48 or 72 h. qRT-PCR using a SYBR green reaction was performed as described previously [23]. RORα and NCEH1 expression levels were measured, with CD11b and MMP9 as positive controls of macrophage-specific genes and BMAL1 as a positive control RORα-target genes. Additionally, the expression of ATGL and LIPE was measured to indicate the expression of genes involved in lipid hydrolysis. These genes were quantified using specific primer sets with the following protocol: 40 cycles at 95 °C for 10 s, 56 °C for 10 s, and 72 °C for 15 s, following initial denaturation at 95 °C for 2 min. The 18S rRNA gene was used as an internal control. Primer sequences for all genes are listed in Supplementary Table S1. Data were collected from at least three independent experiments.

### Western blotting analysis

Cells were washed in phosphate-buffered saline (PBS) and homogenized in lysis buffer containing 50 mM Tris-HCl (pH 7.0), 200 mM sucrose, 1 mM ethylenediaminetetraacetic acid, 1 μg/mL leupeptin, 1 μg/mL pepstatin, 0.5 mM phenylmethylsulfonyl fluoride, and 1% SDS on ice. The samples were subjected to SDS-polyacrylamide gel electrophoresis on 12% gels. Proteins on the SDS-slab gel were transferred to Immobilon-P membranes (Merck Millipore, Billerica, MA, USA) by electrophoresis. Detection of proteins was performed using rabbit anti-RORα (cat. no. sc-28,612; Santa Cruz Biotechnology), anti-NCEH1 (cat. no. PA5–50285; Thermo Fisher Scientific, Waltham, MA, USA), and anti-glyceraldehyde 3-phosphate dehydrogenase (GAPDH) antibodies (cat. no. sc-20,357; Santa Cruz Biotechnology). Subsequently, the membranes were rinsed and incubated with horseradish peroxidase-conjugated goat anti-rabbit IgG. Bound antibodies were detected with enhanced chemiluminescence western blotting detection regents (GE Healthcare) according to the manufacturer’s instructions. The band intensities were analyzed with a CS Analyzer (Atto).

### Overexpression analysis

HEK293 cells were transfected using Lipofectamine 2000 (Life Technologies). Briefly, cells were seeded at 1 × 10^5^ cells/well in D-MEM containing 10% fetal calf serum. After 1 day at 37 °C and 5% CO_2_, cells in each well were transfected for 16 h with a mixture of 500 ng RORα expression plasmid (pRORα) or empty plasmid (pSG5). Cells were grown for an additional 48 h in fresh medium and lysed. The lysate was assayed for qRT-PCR using a SYBR green reaction performed as described previously [23].

### siRNA experiments

siRNAs targeting different sequences in RORα (siRORa-258 and siRORa-1388) were generated using an in vitro transcription T7 kit (Takara Bio). siRNA against green fluorescent protein (siGFP) was used as a negative control. siRNA oligonucleotide sequences are listed in Supplementary Table S1. HepG2 cells were seeded in 24-well plates at 0.5 × 10^5^ cells/well and transfected using Lipofectamine 2000 (Life Technologies) with siRNA the following day. Cells were harvested 48 h after transfection, and total RNA was prepared using ISOGEN reagent (Wako Pure Chemical, Osaka, Japan). RORα and NCEH1 levels were quantified by qRT-PCR as described above. The effects of siRNA transfection on cell viability were estimated by measuring LDH activity using a Cytotoxicity Detection Kit PLUS (Roche), following the manufacturer’s protocol. Data were collected from at least three independent experiments.

### Lipid droplet assays

Oil Red O stock solution was prepared in isopropanol (0.3 g/100 mL). siRNA-transfected HepG2 cells were fixed with 4% paraformaldehyde for 10 min and washed with PBS. The cells were then soaked in 60% Oil Red O stock solution diluted with distilled water for 20 min, and stained cells were washed with PBS. Cells were observed using a Leica TCS-SPE DMI4000B microscope (Leica Microsystems, Wetzlar, Germany). Moreover, lipid droplet accumulation was measured by determining the absorbance at 510 nm in isopropanol-eluted samples from stained cells. Normalization was carried out according to the protein concentrations determined using a protein assay kit (Bio-Rad Laboratories, Hercules, CA, USA).

### Cholesterol content measurement

RAW264.7 cells were seeded at 6 × 10^5^ cells/well into 6-well plates in DMEM supplemented with 10% FBS. After 24 h at 37 °C and 5% CO_2_, RAW264.7 cells were transfected with 1 μg pRORα or pSG5 vector for 24 h. Cells were cultured for 24 h with 10 μg/mL ox-LDL (Thermo Fisher Scientific) for the lipid droplet formation. Subsequently, cells were extracted in a lysis buffer and centrifuged at 1000 g for 10 min. The supernatant was mixed with Folch extract (chloroform–methanol, 2:1), and kept on a shaker for 10 min. Mixture was centrifuged at 3000 g for 10 min, and the supernatant was evaporated by drying up and then dissolved in 200 μL of isopropyl alcohol containing 1% Triton X-100. Total cholesterol contents of free- and ester-types of intracellular cholesterol were measured using Cholesterol E-test Wako (Wako Pure Chemical). Cholesterol levels were normalized to the protein content.

### Statistical analysis

All data are expressed as means ± standard errors unless otherwise stated. Comparisons between two groups were made with unpaired Student’s t-tests. In all cases, results with *P* values of less than 0.05 were considered significant.

## Supplementary information

**Additional file 1: Table S1.** Primers used in this study.

**Additional file 2.** Supplementary figure of Chromatin immunoprecipitation (ChIP) assays (Fig. 2c). ChIP assays were performed using chromatin isolated from human monocytes and differentiated macrophages treated with 100 nM phorbol 12-myristate 13-acetate (PMA) for 24 h. Crosslinked cell lysates were immunoprecipitated with rabbit IgG (IgG) or polyclonal anti-RORα-specific antibodies (RORα). DNA precipitates were isolated and then subjected to PCR using primer pairs covering either RORE1 (fragment size, 253 bp) or RORE2 (fragment size, 247 bp) of the NCEH1 promoter region. Control PCR was performed with non-immunoprecipitated genomic DNA (input). M, size marker.

**Additional file 3.** Supplementary figure of immunoblot analysis (Fig. 5e). THP1 cells were treated with or without 100 nM phorbol 12-myristate 13-acetate (PMA) for 24 h. Protein expression of RORα, NCEH1, and GAPDH was analyzed by immunoblot analysis. Molecular weight of RORα, NCEH1 and GAPDH are 60, 48 and 37 kDa, respectively.

**Additional file 4.** Supplementary figure of immunoblot analysis (Fig. 7d). THP1 cells were treated with 100 nM phorbol 12-myristate 13-acetate (PMA) for 72 h and then treated without or with 5 μM SR1078 for 24 h. Protein expression of NCEH1 and GAPDH was analyzed by immunoblotting. Molecular weight of NCEH1 and GAPDH are 48 and 37 kDa, respectively.

## Data Availability

All data generated or analyzed during this study are included in this article and its supplementary information files.

## Abbreviations

RORα: Retinoic acid receptor-related orphan receptor α
ROREs: RORα response elements
NCEH1: Neutral cholesterol ester hydrolase 1
CE: Cholesterol ester
FC: Free cholesterol
LDLR: Low-density lipoprotein receptor
PGC-1α: Peroxisome proliferator-activated receptor γ coactivator 1-α
TSS: Transcription start site
24-OHC: 24-hydroxycholesterol
APOA: Apolipoprotein A
G6Pase: Glucose 6-phosphatase
PEPCK: Phosphoenolpyruvate carboxykinase
CLDND1: Claudin domain containing 1
PMA: Phorbol 12-myristate 13-acetate
ATGL: Adipose triglyceride lipase
LIPE: Lipase E
LDH: Lactate dehydrogenase
ox-LDL: oxidized low density lipoprotein
NASH: Nonalcoholic steatohepatitis
LXRα: Liver X receptor α
PPARs: Peroxisome proliferator-activated receptors
ER: Estrogen receptor
SRMs: Selective receptor modulators
EMSA: Electrophoretic mobility shift assay
ChIP-PCR: Chromatin immunoprecipitation polymerase chain reaction
qRT-PCR: quantitative reverse transcription-PCR
