# Supplementary material for: Retinoic acid receptor-related orphan receptor α reduces lipid droplets by upregulating neutral cholesterol ester hydrolase 1 in macrophages
Source: BMC Mol Cell Biol. 2020 Apr 22;21:32. doi: 10.1186/s12860-020-00276-z (PMC7310410; doi:10.1186/s12860-020-00276-z)
Supplement: Supplementary file 1 — Additional file 1: Table S1. Primers used in this study. [file 12860_2020_276_MOESM1_ESM.doc]

### **Retinoic acid receptor-related orphan receptor α reduces lipid droplets by upregulating neutral cholesterol ester hydrolase 1** **in macrophages**

### Hiroshi Matsuoka^1, *^ Riki Tokunaga^1^, Miyu Katayama^1^, Yuichiro Hosoda^1^, Kaoruko Miya^1^, Kento Sumi^1^, Ami Ohishi^1^, Jun Kamishikiryo^2^, Akiho Shima^1^, and Akihiro Michihara^1^

### ^1^Laboratory of Genome Function and Pathophysiology, Faculty of Pharmacy and Pharmaceutical Sciences, Fukuyama University, Fukuyama, Hiroshima 729-0292, Japan

### ^2^Laboratory of Biochemistry, Faculty of Pharmacy and Pharmaceutical Sciences, Fukuyama University, Fukuyama, Hiroshima 729-0292, Japan

### * Corresponding author

### E-mail: [*matsuoka@fukuyama-u.ac.jp*](mailto:matsuoka@fupharm.fukuyama-u.ac.jp) (HM)

**Supplementary Table S1.** Primers used in this study.

Primer Sequence (5′–3′)

***EMSA*** (Mutated sequences are underlined)

NCEH1-RORE1wt-sense GGGCAGATCACAAGGTCAGGAGTT

NCEH1-RORE1wt-antisense AACTCCTGACCTTGTGATCTGCCC

NCEH1-RORE2wt-sense TCAGAATGACCCACTTGCTTTGTC

NCEH1-RORE2wt-antisense GACAAAGCAAGTGGGTCATTCTGA

NCEH1-RORE1mt-sense GGAAATAGGATCCGTCAACGTT

NCEH1-RORE1mt-antisense AACGTTGACGGATCCTATTTCC

NCEH1-RORE2mt-sense TCAGAATGACGGACCCGCTTTGTC

NCEH1-RORE2mt-antisense GACAAAGCGGGTCCGTCATTCTGA

Ikb-RORE-sense GATCCAATGTAGGTCACATG

Ikb-RORE-antisense CATGTGACCTACATTGGATC

***ChIP-PCR***

ChIP-NCEH1-RORE1-FW CCAGGTGCCGTGGATCATGC

ChIP-NCEH1-RORE1-RV GGCTAGAGTGCAATGGCACG

ChIP-NCEH1-RORE2-FW TTCATTGCTTGAGCCAGAAG

ChIP-NCEH1-RORE2-RV GCGGACTCTGGAGGACACTG

***Luciferase reporter cloning*** (Mutated sequences are underlined)

pNCEH1(-1689)-KpnI-FW CCTGGGTACCAAGAGCGATGCTCTATCTC

pNCEH1(+128)-MluI-RV CATTCACGCGTTTCTTATAAGCCCAGGAGC

pNCEH1(-140)-wt-KpnI-FW CAGTGGTACCAGTCAGAATGACCCACTTGC

pNCEH1(-140)-mt-KpnI-FW CAGTGGTACCAGTCAGAATGACGGACCCGCTTTGTCCAG

PGVB2-FW GCCCAAGCTACCATGATAAG

PGVB2-RV TCATAGCTTCTGCCAACCGAAC

***qRT-PCR***

rtRORA-FW TCCATGCAAGATCTGTGGAG

rtRORA-RV ACAGCATCTCGAGACATCCC

rtNCEH1-FW TGTGTACAGCAATGGCTGAG

rtNCEH1-RV CTTCTGGCTTCAGGAAATAC

rtBMAL1-FW GTTCTTCTATTCTTGGTGAGAAC

rtBMAL1-RV ACAGCCATTGCTGCCTCATC

rtCD11-FW GGATGACCTCAGCATCACCT

rtCD11-RV GGTTCTGGGCATGTTGTTCT

rtMMP9-FW GCCTGCAACGTGAACATCT

rtMMP9-RV TCAAAGACCGAGTCCAGCTT

rtLIPE-FW CAGGCTCATCTCCTATGACCTGCG

rtLIPE-RV CGTGGAAGTGCACTATCAGGGACC

rtATGL-FW GCTCACGGCCACGGCGCTGGTCAC

rtATGL-RV TCAGCAGGCAGGACCTTCAGCAGG

rt18S rRNA-FW CGATAACGAACGAGACTCTGG

rt18S rRNA-RV TAGGGTAGGCACACGCTGAGC

***SiRNA experiments*** (Synthesised siRNA sequences are underlined)

siRORA258-S-u GATCACTAATACGACTCACTATAGGGGTCAGAAGAACTGTTTGATTT

siRORA258-S-d AAATCAAACAGTTCTTCTGACCCCTATAGTGAGTCGTATTAGTGATC

siRORA258-AS-u GATCACTAATACGACTCACTATAGGGATCAAACAGTTCTTCTGACTT

siRORA258-AS-d AAGTCAGAAGAACTGTTTGATCCCTATAGTGAGTCGTATTAGTGATC

siRORA1388-S-u GATCACTAATACGACTCACTATAGGGCTAATGGCATTTAAAGCAATT

siRORA1388-S-d AATTGCTTTAAATGCCATTAGCCCTATAGTGAGTCGTATTAGTGATC

siRORA1388-AS-u GATCACTAATACGACTCACTATAGGGTTGCTTTAAATGCCATTAGTT

siRORA1388-AS-d AACTAATGGCATTTAAAGCAACCCTATAGTGAGTCGTATTAGTGATC

siGFP-S-u GATCACTAATACGACTCACTATAGGGCAAGCTGACCCTGAAGTTCTT

siGFP-S-d AAGAACTTCAGGGTCAGCTTGCCCTATAGTGAGTCGTATTAGTGATC

siGFP-AS-u GATCACTAATACGACTCACTATAGGGGAACTTCAGGGTCAGCTTGTT

siGFP-AS-d AACAAGCTGACCCTGAAGTTCCCCTATAGTGAGTCGTATTAGTGATC
