# Supplementary figures and images for: Retinoic acid receptor-related orphan receptor α reduces lipid droplets by upregulating neutral cholesterol ester hydrolase 1 in macrophages
Source: BMC Mol Cell Biol. 2020 Apr 22;21:32. doi: 10.1186/s12860-020-00276-z (PMC7310410; doi:10.1186/s12860-020-00276-z)

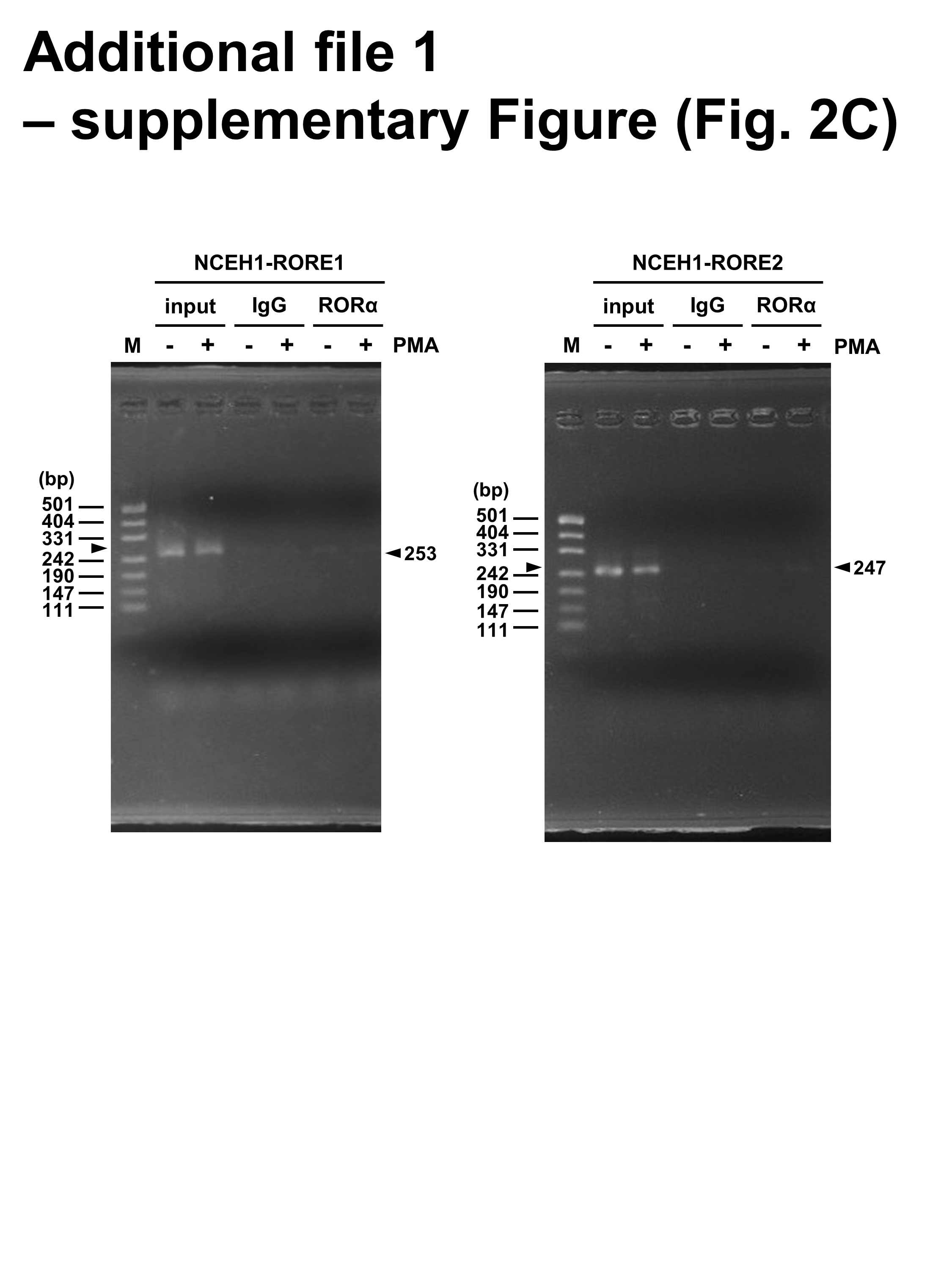

Supplement: Supplementary file 2 — Additional file 2. Supplementary figure of Chromatin immunoprecipitation (ChIP) assays (Fig. 2c). ChIP assays were performed using chromatin isolated from human monocytes and differentiated macrophages treated with 100 nM phorbol 12-myristate 13-acetate (PMA) for 24 h. Crosslinked cell lysates were immunoprecipitated with rabbit IgG (IgG) or polyclonal anti-RORα-specific antibodies (RORα). DNA precipitates were isolated and then subjected to PCR using primer pairs covering either RORE1 (fragment size, 253 bp) or RORE2 (fragment size, 247 bp) of the NCEH1 promoter region. Control PCR was performed with non-immunoprecipitated genomic DNA (input). M, size marker. [file 12860_2020_276_MOESM2_ESM.tif]

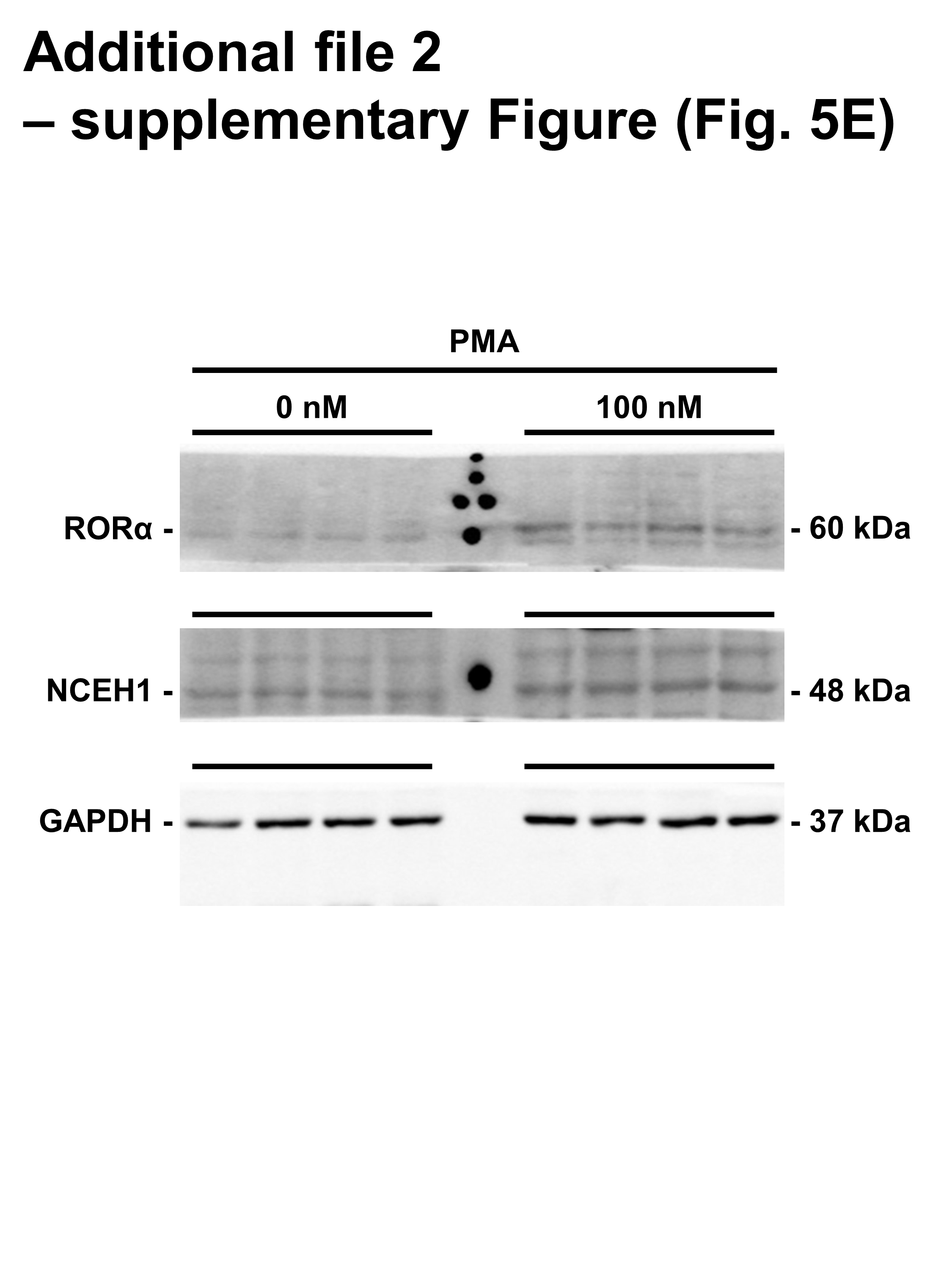

Supplement: Supplementary file 3 — Additional file 3. Supplementary figure of immunoblot analysis (Fig. 5e). THP1 cells were treated with or without 100 nM phorbol 12-myristate 13-acetate (PMA) for 24 h. Protein expression of RORα, NCEH1, and GAPDH was analyzed by immunoblot analysis. Molecular weight of RORα, NCEH1 and GAPDH are 60, 48 and 37 kDa, respectively. [file 12860_2020_276_MOESM3_ESM.tif]

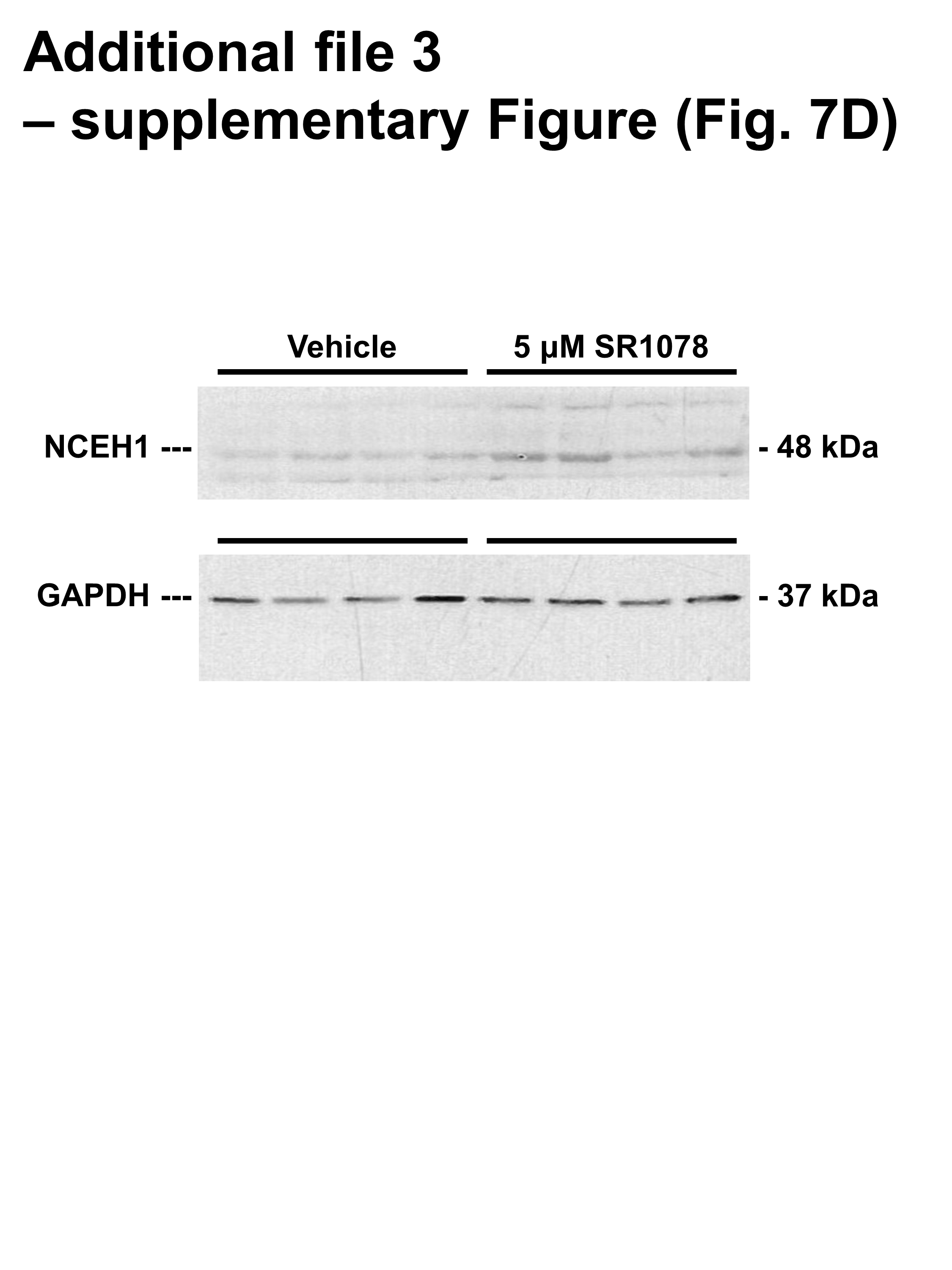

Supplement: Supplementary file 4 — Additional file 4. Supplementary figure of immunoblot analysis (Fig. 7d). THP1 cells were treated with 100 nM phorbol 12-myristate 13-acetate (PMA) for 72 h and then treated without or with 5 μM SR1078 for 24 h. Protein expression of NCEH1 and GAPDH was analyzed by immunoblotting. Molecular weight of NCEH1 and GAPDH are 48 and 37 kDa, respectively. [file 12860_2020_276_MOESM4_ESM.tif]
